# Patient preferences and treatment safety for uncomplicated vulvovaginal candidiasis in primary health care

**DOI:** 10.1186/1471-2458-11-63

**Published:** 2011-01-31

**Authors:** Isabel Del-Cura González, Francisca García-de-Blas González, Teresa Sanz Cuesta, Jesús Martín Fernández, Justo M Del-Alamo Rodríguez, Rosa A Escriva Ferrairo, M del Canto De-Hoyos Alonso, Laura Balsalobre Arenas, Ricardo Rodríguez Barrientos, Elisa Ceresuela Wiesmann, Cristina De-Alba Romero, Yolanda Ginés Díaz, Ana Pastor Rodríguez-Moñino, Blanca Gutiérrez Teira, Marta Sánchez-Celaya del Pozo, Jesús Fernández Horcajuelo, María J Rojas Giraldo, Paulino Cubero González, Rocío A Vello Cuadrado, Beatriz López Uriarte, Jeannet Sánchez Yepes, Yolanda Hernando Sanz, M José Iglesias Piñeiro, Susana Tudanca Hernández, Fernando Gallardo Alonso, Ana I González González, Alicia Simón Fernández, Carmen Carballo, Ana Rey López, Fernanda Morales, Dolores Martínez López

**Affiliations:** 1 Unidad de Investigación. Atención Primaria Area 9. Servicio Madrileño de Salud. Facultad de Ciencias de la Salud, Universidad Rey Juan Carlos, Madrid, Spain; 2 CS. Mendiguchia. Atención Primaria Area 9. Servicio Madrileño de Salud. Spain; 3 Técnico de Salud. Atención Primaria Area 9. Servicio Madrileño de Salud. Spain; 4 Departamento de Medicina de Familia. Atención Primaria Area 8. Servicio Madrileño de Salud. Spain; 5 Servicio de Microbiología. Hospital Severo Ochoa. Leganés. Madrid. Spain; 6 CS Palomares. Atención Primaria Area 9. Servicio Madrileño de Salud. Spain; 7 CS Laín Entralgo. Atención Primaria Area 8. Servicio Madrileño de Salud. Spain; 8 CS Mendiguchia. Atención Primaria Area 9. Servicio Madrileño de Salud. Spain; 9 Unidad de Investigación. Atención Primaria Area 5. Servicio Madrileño de Salud. Spain; 10 CS Palacio de Segovia. Atención Primaria Area 7. Servicio Madrileño de Salud. Spain; 11 CS San Fermín. Atención Primaria Area 11. Servicio Madrileño de Salud. Spain; 12 CS Puerta Bonita. Atención Primaria Area 11. Servicio Madrileño de Salud. Spain; 13 Departamento de Calidad. Atención Primaria Area 11. Servicio Madrileño de Salud. Spain; 14 CS El Soto. Atención Primaria Area 8. Servicio Madrileño de Salud. Spain; 15 Departamento de Medicina de Familia. Atención Primaria Area 1. Servicio Madrileño de Salud. Spain; 16 CS Panaderas. Atención Primaria Area 9. Servicio Madrileño de Salud. Spain; 17 CS Loranca. Atención Primaria Area 9. Servicio Madrileño de Salud. Spain; 18 CS General Ricardos. Atención Primaria Area 11. Servicio Madrileño de Salud. Spain; 19 CS Humanes. Atención Primaria Area 9. Servicio Madrileño de Salud. Spain; 20 CS Pedroches. Atención Primaria Area 9. Servicio Madrileño de Salud. Spain; 21 CS Federica Montseny. Atención Primaria Area 9. Servicio Madrileño de Salud. Spain; 22 CS Vicente Soldevilla. Atención Primaria Area 9. Servicio Madrileño de Salud. Spain; 23 CS Mendiguchia. Atención Primaria Area 9. Servicio Madrileño de Salud. Spain; 24 CS Vicente Muzas. Atención Primaria Area 9. Servicio Madrileño de Salud. Spain; 25 CS Huerta de los Frailes. Atención Primaria Area 9. Servicio Madrileño de Salud. Spain; 26 CS la Fortuna. Atención Primaria Area 9. Servicio Madrileño de Salud. Spain; 27 CS Jaime Vera. Atención Primaria Area 9. Servicio Madrileño de Salud. Spain

## Abstract

**Background:**

Vaginitis is a common complaint in primary care. In uncomplicated candidal vaginitis, there are no differences in effectiveness between oral or vaginal treatment. Some studies describe that the preferred treatment is the oral one, but a Cochrane's review points out inconsistencies associated with the report of the preferred way that limit the use of such data. Risk factors associated with recurrent vulvovaginal candidiasis still remain controversial.

**Methods/Design:**

This work describes a protocol of a multicentric prospective observational study with one year follow up, to describe the women's reasons and preferences to choose the way of administration (oral vs topical) in the treatment of not complicated candidal vaginitis. The number of women required is 765, they are chosen by consecutive sampling. All of whom are aged 16 and over with vaginal discharge and/or vaginal pruritus, diagnosed with not complicated vulvovaginitis in Primary Care in Madrid.

The main outcome variable is the preferences of the patients in treatment choice; secondary outcome variables are time to symptoms relief and adverse reactions and the frequency of recurrent vulvovaginitis and the risk factors. In the statistical analysis, for the main objective will be descriptive for each of the variables, bivariant analysis and multivariate analysis (logistic regression).. The dependent variable being the type of treatment chosen (oral or topical) and the independent, the variables that after bivariant analysis, have been associated to the treatment preference.

**Discussion:**

Clinical decisions, recommendations, and practice guidelines must not only attend to the best available evidence, but also to the values and preferences of the informed patient.

## Background

Vaginitis is the most frequent reason for gynecology consultation in primary health care services. It is estimated that 75% of women experience at least one episode of vulvovaginal candidiasis throughout her life and 40-50% of them have at least one recurrence [1].

The most frequent cause of vulvovaginal inflammation is infective, beeing the main organisms: *Gardnerella vaginalis *(15-50%), *Candida *(C) (20-25%) and *Trichomonas vaginalis *(5-50%) species, with a frequency distribution that depends on the populations studied [2]. Candidal vaginitis is a generic term used for vaginal infections caused by *Candida *species. *Candida albicans *is responsible for 90% of vulvovaginal candidiasis; the remaining 10% corresponds to *C. glabrata *and *C. tropicalis *[2,3]. No data are available for this distribution in our setting.

Microscopic examination of vaginal discharge is the main tool for diagnosis. It allows the diagnosis in most of the cases. Fungal culture is useful when the mycroscopic examination of the swab is negative and patient refers suggestive symptoms of Candida infection or in cases of chronic recurrent vulvovaginitis. The culture is not useful in women who received an anti-fungal therapy during last week (90% will have a negative culture) [3]. Common practice is, when symptoms of suspicion occur, to take a clinical history, physical examination and a vaginal swab, and initiating empirical treatment before microbiological confirmation. However, symptoms alone do not allow clinicians to distinguish confidently between the causes of vaginitis. The sensitivity of the classic symptoms of vulvovaginal candidiasis (itching, white cheesy discharge) ranges between 41 and 91% and a specificity between 47 and 73%; this is why the microbiological study is recommended [4].

Vulvovaginal candidiasis is treated with a variety of anti-fungal drugs, administered oral or topically (vaginal) [5-9]. In simple or uncomplicated cases there is no difference in the relative effectiveness (measured as clinical and mycological cure) of anti-fungals in both ways of administration (vaginal and oral), including single-dose regimens, neither between preparations of different time and dose intervals (level 1 of evidence). Patient preferences, the response to previous treatment and the cost should guide our choice [7].

The main purpose of Cochrane's review (2007), which included 17 clinical trials, was to assess the relative effectiveness of anti-fungals imidazol y triazol (both oral and intravaginal) for the treatment of uncomplicated vaginal candidiasis. Secondary endpoints evaluated the cost-effectiveness, safety and patient preference of oral versus intravaginal anti-fungals. According to this review no definitive conclusion can be made regarding the relative safety of oral and intravaginal anti-fungals for uncomplicated vaginal candidiasis because side effects data were poorly reported in different studies. It should be noted that in this review trials involving ketoconazol due to "its association with serious adverse reactions and to its limited license" were excluded [8].

Intravaginal anti-fungals present a higher frequency of topical reactions (eg irritation, burning, itching) than the ones administered orally, although systemic effects (headache) are also reported. The oral administration is associated with a wide range of systemic effects including gastrointestinal side effects and headache. The rate of reported side effects per 100 patients was for each anti-fungal the follow: 21% for fluconazol, 22% for clotrimazol, 23% for itraconazol and 12% for econazol and miconazol [8].

Ten clinical trials dealt with women's preference regarding the anti-fungals way of administration, but the presented data were not sufficient to answer this question [10-20]. The Cochrane review's authors concluded that all studies that dealt with preferences are in favor of oral treatment (compared to intravaginal or no preference) with a fluctuation from 43% for women at Van Heusdenal's trial [20] to 93% at Timonen's trial [16]. Nevertheless, inconsistencies associated with the report of the preferred way of administration limit the use of such data. According Cochrane review, at the studies dealing with preferences, patients are inclined to oral treatment against vaginal between 46% [18] and 93% [16] of the cases.

We should bear in mind, however, that oral preparations are generally more expensive than intravaginal treatments and systemic side effects associated with oral therapy are likely to be more serious than the intravaginal one [21]. As for clinical implications the authors of the review suggest that, unless there is a history of adverse reaction to one of the ways of administration or contraindications, women who acquire their own treatment should receive full information regarding the characteristics and cost of treatment, allowing them to make their own decision. If health services are paying the treatment cost, decision-makers should consider whether the higher cost of some oral anti-fungals assume is worth the gain in convenience, if this is the patient's preference [8].

According to american statistics between 5% -7% of women will have recurrent candidal vulvovaginitis (VVCR), defined as 4 or more microbiologically documented episodes in a year. In these cases, although *C. glabrata *was isolated in a 15% ratio, *C. albicans *is still the most common responsible organism [2]. There are several pathophysiological theories. Some authors have described an alteration of topical immunity with a disproportionate response to small amounts of antigen of *Candida *mediated through IgE [2,22]. But there is controversy about some of the causes that predispose to vaginal colonization. Other causes described in the medical literature are: increased estrogen levels (pregnancy, especially the third quarter; high-dose oral contraceptives; the luteal phase of menstrual cycle), recent treatment with broad spectrum antibiotics (ampicillin, tetracyclines and cephalosporins), situations of immunosuppression (treatments with corticosteroids, HIV) and diabetes mellitus with poor control [23,24]. Although clinical experience highlights a relationship between the presence of candidal vaginitis and the use of contraceptives, this still remains controversial.

Clinical decisions, recommendations, and practice guidelines must not only attend to the best available evidence, but also to the values and preferences of the informed patient [25]. Identifying, critically appraising, and summarizing the evidence were initial areas of focus for evidence-based medicine (EBM). However, evidence alone is not sufficient to make clinical decisions [26]. In 2000, the EBM Working Group presented the second fundamental principle of EBM (the hierarchy of evidence being the first): whatever the evidence, value and preference judgments are implicit in every clinical decision. Values and preferences refer not only the patients' perspectives, beliefs, expectations, and goals for life and health, but also the processes individuals use to consider the available options and their relative benefits, harms, costs, and inconveniences.

In the methodological manuals for the development of clinical practice guidelines, the incorporation of the patient's perspective is considered. In order to achive this, the following methodological options are proposed: a review of the literature on the subject from the patient's perspective; investigation focused on the perspective of patients; and the inclusion of patients in the process of development and discussion of the draft guide [27-30]

In order to develop researches that allow to incorporate patient's preferences in medical-decisions making, the different settings of decisions must be considered. The first field of decision is individual (micro level) and it should include decisions such as choice of doctor, how to provide information or choice of treatment. The second level (meso) is related to clinical decision guidelines for patient groups with similar characteristics and requires the combination of different preferences (groups of people with different preferences). The third level is called macro or social level. It deals essentially with elections of health programs and economic aspects and should always be taken into account [31].

Our research proposal focuses on the micro level of decision making, studying preferences in individual decision making. The results can also be used at the meso level, through their inclusion into clinical practice guidelines. Our intention is to answer the pending questions that the Cochrane review authors highlight at the *implications for research *section [8].

The main aim of our study is to describe the preferences of women and the factors that let them to choose the type of administration (oral or topical) for the treatment of uncomplicated vulvovaginal candidiasis in primary health care. Secondary objectives are to determine: 1) the prevalence of different candida species, 2) the safety of administered treatments, calculated as the number of topical or systemic adverse reactions, 3) time of symptom relief and 4) the frequency of recurrent vulvovaginal candidiasis (> 4 episodes per year, documented microbiologically) as well as predisposing factors.

## Methods/Design

### Design of study

Prospective observational study of one year follow up. Post-authorization study

### Setting

17 public health centers, Primary Care Health Service of Madrid (Spain).

### Type of participants

**Figure 1:**
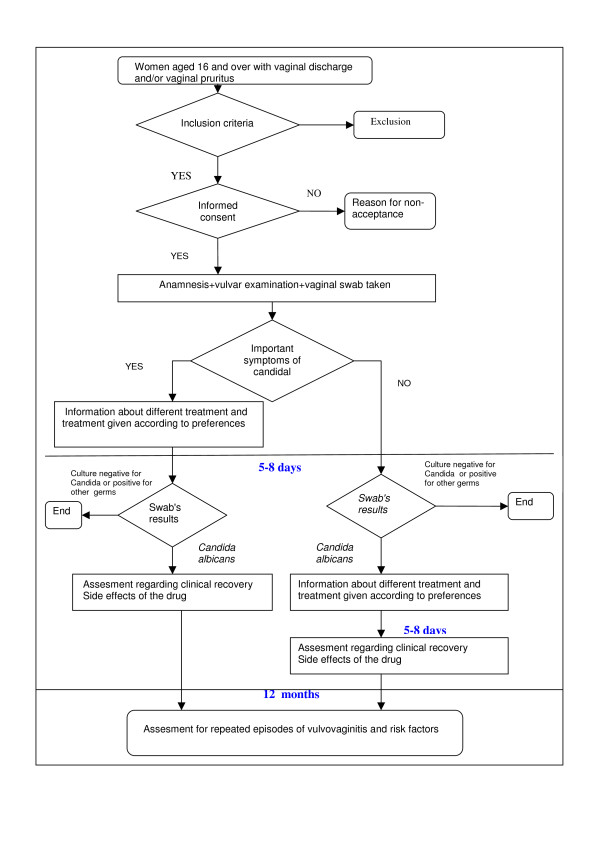
**Trial profile**.

Women aged 16 years or over with symptoms of vaginitis (see Figure 1)

#### Criteria of exclusion

1. Pharmacological allergy to any of the proposed treatments.

2. Women with complicated candidiasis (pregnancy, symptoms or severe inflammation, no presence of Candida-albicans species, recurrence, poorly controlled diabetes mellitus).

3. Treatment with topical or oral antifungals in the week prior to the consultation.

4. Patients who are away from their homes and do not intend to reside in the district of the health service in the following year.

5. Patients were the taking of the swab is not possible.

6. Having had sex without use of barrier methods in the previous 24 hours.

7. Intravaginal showers in the last 12 hours.

8. Breastfeeding.

9. Use of oral corticosteroids or immunosuppressants in the last week.

10. HIV.

11. Patients taking oral anticoagulants or anticonvulsants.

11. Mental illnesses that prevent data collection.

### Sample size

It was calculated to estimate a proportion, assuming an expected preference for oral treatment of 43% with an alpha risk of 0.05 and a precision 0.4. The sample size was overestimated, considering a 20% loss. The sample size required is 703.

### Sampling technique

Assistant researchers included in the study all patients who meet the selection criteria during the year-long recruitment. Patients will be captured in a total of 17 Primary Health Care Centres of the National Health Service by 21 family physician.

### Variables

The variable result will be *choice of treatment*: type of drug, oral or intravaginal administration (pessary or cream), single or multiple doses and reasons for choosing it. The independent variables, will be: the demographics (age, nationality, employment status, educational level, family income per month), data from the history (history of vaginal candidiasis, previous experience of treatment, use of vaginal antiseptics), side effects (present side effects in the first week, treatment discontinuation due to side effects), time to symptoms disappearance, and etiologic (*Candida *species).

To study the factors associated with recurrent vulvovaginal candidiasis, the variables are: number of episodes of vaginitis per year (documented microbiologically), the use of hormonal contraceptives, hormone replacement therapy, allergic rhinitis, diabetes mellitus, antibiotic administration in the last week (specifying the type of antibiotic), use of spermicides, panty or pantyliners, intravaginal showers, history of childbirth, menstrual phase and sex.

### Data collection

The information will be collected by the patient's own family physician through a personal interview. In patients who met the inclusion criteria, the physician will inform the patient of the study's characteristics and will ask informed consent. Women who do not agree to participate will be asked why.

During first visit anamnesis and vulvar inspection will be carried out, with vaginal swab taking. A cotton swab transport medium will be used, that will be sent to the reference laboratory within 24 hours, as standard practice. In women with few symptoms the start of the treatment may be delayed until the microbiological confirmation.

The doctor will inform the patient about treatment options (drugs, administration, dosages and costs) and a treatment will be initiated according to her preference. The proposed prescription options are the ones recommended in the literature [2,6,9,21]. The information provided to women has been developed taking into account the results of a discussion group of women with candidal vaginitis and primary health care physicians working on different formats and content. The final proposal includes three cards that provide various options grouped by: oral/vaginal administration, single dose/multi dose, side effects and costs.

The second visit will take place in a period between 5-8 days after initiating treatment. Microbiological diagnosis will be confirmed. Clinical cure (disappearance of symptoms) will be assessed as well as possible side effects.

If no treatment was started at the first visit, information on treatments will be offered to woman and preferences will be asked. In these cases, information on side effects and symptoms disappearance after 7 days will be gathered by telephone.

Patient will be informed for the necessity of contacting the doctor at the appearance of new episodes.

The third visit will be arranged after the review of the clinical history, 12 months later. Whenever is possible, patient will be given an appointment in order to gather information; if it is not possible, the consultation will be carried out by telephone.

For this study an electronic notebook for data collection was designed.

### Type of analysis

Will be described for each of the objectives.

#### Main objective

Women's preferences on way of administration will be described, by calculating proportions with the corresponding confidence intervals. Thereafter, the association of each of the independent variables to the dependent ones will be studied, using as statistical tests to compare proportions Pearson's chi-square test or Fisher's exact test (when chi-square test conditions of application are not satisfactory) and to compare means Student's t-test or Mann Whitney U nonparametric test.

Afterwards, multivariate analysis will be carried out, in order to build an explanatory model, where the dependent variable is the chosen way of administration, and the independent variables, the ones that in the bivariate analysis were associated with patient's preference or such an association is described in the literature.

#### Secondary objectives

##### Objective 1

To describe the Candida species responsible of the clinic

by proportions-calculator with their confidence intervals.

##### Objective 2

To describe the adverse reactions observed by using the mean and standard deviation. To compare the adverse reactions for treatment regarding the way of administration, Student's t-test or Mann Whitney U non-parametric test will be used.

##### Objective 3

To calculate the average number of days in the relief of symptoms for each way of administration. A survival analysis will be carried out applying Cox's regression model.

##### Objective 4

To calculate the frequency of patients with VVCR criteria. There will be a multivariate analysis, applying logistic regression: the dependent variable will be recurrence (VVCR criteria) and the independent variables the ones that in the bivariate analysis were associated with the recurrence of infection or are described in the literature as risk factors.

### Limitations of the study

Patients will be included in the study by their own family physicians. The implication of a large number of professionals will increase diagnosis variability (in this case limited beeing a microbiological diagnosis).

The microbiological study will be carried out by 5 reference laboratories, therefore variability in the methods of sample analysis might also increase. But then, the results will reflect what is done usually in everyday-practice which gives the study feasibility and applicability.

Doctor's style and his preferences can influence the way in which different treatment alternatives are offered to the patient, so bias might be introduced. This will be minimized by the choice card system of preferences in which all participant doctors will be trained in a 4 hours session.

Although we have to contemplate losses in the follow up process, in primary health care service this is reduced due to the total accessibility to the public health system. In order to give answer to our main objectives, the follow up is of 7 days and it is unlikely that losses will be important. However, with the purpose of minimize them, women who do not attend the scheduled visit, will be located by telephone and try to recapture them. If she is unable to attend the consultation, the information on side effects will be gathered by telephone. The losses after a year may be higher but we will try to reduce them in the same way. Patients consent to be included in the study and the fact that her own family physician will make this offer, will limit losses as it is described in previous studies [32,33]. Although the ideal design for comparing the safety of various treatments is the clinical trial, post-authorization observational studies are also of interest, in order to describe side effects, especially by making use of the information gathered in usual clinical practice.

### Ethical considerations

The study has been approved by the Clinical Research Ethics Committee of Area 9 of the Primary Care of Madrid. It has been registered with reference code ICG/TVC/2008/01 and it has also been authorized by the Department of Health of the Community of Madrid. The study will respect the 2008 Helsinki Declaration. Confidentiality and anonymity of data will be strictly maintained, according to 15/1999 data protection act, both in the implementation phase of the project and in the resulting presentations or publications. Any suspected adverse reaction to treatment will be reported to the Pharmacovigilance Centre of the Community of Madrid.

## Discussion

Our study aims to provide information about the preferences of patients and the safety of treatments in order to help the family physician to take the decision to prescribe or recommend an oral or intavaginal antimycotic. To make this decision within the health system, beside efficacy and safety of treatment, costs and patient preferences, must be also taken into account. These preferences are mostly studied by the pharmaceutical industry on their own products, but in populations highly different from the Spanish one.

The choice from various medical treatments is not a purely technical problem. As suggested by some authors, these elections call to resolve dilemmas that are emotive and subjective. Situations that are not usual during consultations but are normal when choosing a car or in different personal decision taking. Patients are not accustomed to these dilemmas in relation to health and illness and can not make these decisions intuitively. We need to make these kind of decisions in a structured way [31]

It is essential that treatments in clinical practice guidelines should incorporate the results of researches which main objective is to study the patient's preferences. In recent years, all agencies that develop guidelines are preparing methodological proposals in this regard [27,29]. Our proposed methodology has a quantitative approach but it could be expanded in a second phase also with a qualitative approach that explore other areas related to patient preferences, and allows to benefit from both methodologies [34].

The fact that the aim of the study was the safety of treatments had determined its characteristics. Initially we designed a prospective observational study that would reflect the preferences of patients, but adverse effects of treatments was a question still unanswered. By including such an aspect, our study became a post-authorization study with medication, although all treatments offered are licensed drugs, according the legislation of the Community of Madrid, with a longstanding experience.

From the clinical point of view, the study incorporates some new aspects to measure therapeutic response. In most drug-efficacy studies, clinical cure is assessed after 7 days [12,17,18,20], and after 5-16 days [11,35,36] but we think it is interesting to evaluate the time for the relief of symptoms, since there is disagreement about whether the symptoms were relieved earlier with oral or topical drugs. In this way, Seidman LS [37] reported that the relief of symptoms is 24 hours with topics and 46 hours with orals. However, another study [11] reports that time is shorter with oral fluconazole (1 day) in comparison to topical chlotrimazole (2 days).

On the other hand, risk factors associated with recurrent vulvovaginal candidiasis still remain controversial [23] and an approach from a prospective study like the one proposed here can help answering this question.

As said before vaginitis is the most frequent gynecological cause of consultation in primary health care and many women throughout their life will consult their doctors for this reason. The different treatment options have all limited side effects, only exceptionally severe ones, and cost rates are affordable to the majority of the population.

Probably all agree on the importance of incorporating preferences in dilemmas with a high emotional charge (cancer treatments, surgeries, etc). But we can not ignore the fact that although family physicians in many cases have to inform the patient for serious health problems or for processes that limit their quality of life, there are many more everyday clinical decisions for minor health problems that are very important.

It is important to raise research questions that can help us to make progress in the incorporation of patient preferences to the decision-making, so we can contribute to the change of the doctor-patient relationship. JL Pinto y cols [31] quote professor Alan Williams in his ingenious play on words where "the patient is to give the doctor all the information the physician deems necessary, so the doctor can make a decision and the patient should do it once the doctor has taken the decision" has changed to a relationship where "the physician is to give the patient all the information the patient deems necessary for the patient to take a decision, and the physician should do it once the patient has taken the decision".

## Competing interests

The authors declare that they have no competing interests.

## Authors' contributions

ICG conceived the study, participated in de design, coordinated, and reviewed the draft and the final manuscript. FGB coordinated the survey and reviewed the draft and the final manuscript. TSC participated in the design, coordinated the authorization, and reviewed the draft and the final manuscript. JMF, MAR, REF, CHA, LBA, RRB, ECW, CAR, YGD, APR, BGT, MSC, participated in the design of the study, and reviewed the manuscript. The another authors of PRESEVAC group review the final manuscript.

## Pre-publication history

The pre-publication history for this paper can be accessed here:

http://www.biomedcentral.com/1471-2458/11/63/prepub
